# Supplementary material for: Chromothripsis during telomere crisis is independent of NHEJ, and consistent with a replicative origin
Source: Genome Res. 2019 May;29(5):737–49. doi: 10.1101/gr.240705.118 (PMC6499312; doi:10.1101/gr.240705.118)
Supplement: Supplemental Material [file supp_gr.240705.118_Supplemental_file_1.zip › contigs/annotated_contigs/DB68/contig.4.DB68_length_586_mean_cov_16.5392491468.docx]

**DB68_length_586_mean_cov_16.5392491468**

```
AAAATAATAAAAACAAGGGAGAATGAAGAGTCAGGTACGTAAACTTTCTGAAGCATTTTTCTTTTTTCAAAT|AC|CTGAGACTGGGTA
 >chr7:109084504-109084576 - E=1e-31                                       >chr1:661166
ATTTATAAAGAAAAGAGGTTTAATTGGCTCATGGTTCCACAGGCTGTACAGGAAGCATGACGGCTTCTGAGGAGG|TCCCTCCCAGG|A
02-66116690 - E=3e-29
GACTTTCTTTTTCTTTTCTTTTCTTTTCTT|TTTTTTTTTTTTT|TGAGATGGAGTTTCACTCTTGTCACCCAGGCTGGAGTGCAGTGG
>chr12:12580087-12580131 + E=9e-06 p=0e+00 >chr7:109085602-109085983 + E=3e-177
CGTGATCTCGGCTCACTGCAAGCTCCGCCTCCCGGGTTCATGCCATTCTCCTGCCTCAGCCTCCTGAGTAGCTGGGACTACAGGCGCCC

GCCACCACGCCCGGCTAATTTTTTGTATTTTTAGTAGAGACGGGGTTTCACCAGGATGGTCTCGATCTCTGACCTCGTGATCTGCCCGC

CTCGGCCTCCCAAAGTGCTGGGATTACAGGCGTGAGCCACCGCGCCTGGCCCTACCTCTTTGTTTTCTTACAAGCAAAGGTAACTTTCC

AGTGCAGACATTTTCTCCTTGTATTTACACTTTAATGTTATATTTGTACAATTGATGT
```
